# The exercise-app Axia for axial spondyloarthritis enhances the home-based exercise frequency in axial spondyloarthritis patients – A cross-sectional survey

**DOI:** 10.1007/s00296-024-05600-w

**Published:** 2024-04-29

**Authors:** Patrick-Pascal Strunz, Maxime Le Maire, Tobias Heusinger, Juliana Klein, Hannah Labinsky, Anna Fleischer, Karsten Sebastian Luetkens, Patricia Possler, Michael Gernert, Robert Leppich, Astrid Schmieder, Ludwig Hammel, Evelin Schulz, Billy Sperlich, Matthias Froehlich, Marc Schmalzing

**Affiliations:** 1 Department of Internal Medicine 2, Rheumatology/Clinical Immunology, University Hospital Würzburg, Oberdürrbacher Straße 6, 97080 Würzburg, Germany; 2 Medical Faculty, University of Würzburg, Josef-Schneider-Straße 2, 97080 Würzburg, Germany; 3 Department of Internal Medicine 2, Psychosomatic Medicine, University Hospital Würzburg, Oberdürrbacher Straße 6, 97080 Würzburg, Germany; 4 Department of Diagnostic and Interventional Radiology, University Hospital Würzburg, Oberdürrbacher Straße 6, 97080 Würzburg, Germany; 5 Chair of Software Engineering (Informatik II), Department of Computer Science, University of Würzburg, Am Hubland, 97074 Würzburg, Germany; 6 Department of Dermatology, Venereology, and Allergology, University Hospital Würzburg, Josef-Schneider-Straße 2, 97080 Würzburg, Germany; 7 Deutsche Vereinigung Morbus Bechterew e. V, Metzgergasse 16, 97421 Schweinfurt, Germany; 8 Integrative and Experimental Exercise Science and Training, Institute for Sports Science, University of Wuerzburg, Judenbühlweg 11, 97082 Würzburg, Germany

**Keywords:** Mobile Health Units, Physical therapy modalities, Spondylitis Ankylosing, Patient education as topic, Surveys and questionnaires

## Abstract

Background: Patients with axial spondyloarthritis (axSpA) benefit from regular home-based exercise (HbE). In spite of recommendations, a relevant proportion of German axSpA patients does not adhere to recommended HbE practices. To enhance HbE care, we developed the novel digital therapeutic (DTx) “Axia” compliant with the European medical device regulation (MDR). Axia offers a modern app-based HbE solution with patient educative content and further integrated features. Objective: We aimed to assess Axia’s efficacy, attractiveness, and functionality through a survey among axSpA-patients involved in the first user tests. Methods: A mixed-method online questionnaire with 38 items was administered to 37 axSpA volunteers after using Axia. Numeric rating scales (NRS) and likelihood scales were primarily used. Results: HbE frequency significantly increased from a median of 1 day/week to 6 days/week (*p* < 0.001) by using Axia. Existing HbE practitioners also increased their frequency (median of 4 days/week before, 6 days/week with Axia, *p* < 0.05). Axia received a median rating of 5 out of 5 stars. On NRS scales, Axia scored a median of 9 for intuitiveness and design, and a median of 8 for entertainment. 64.9% reported improved range of motion, 43.2% reported reduced pain, and 93.6% enhanced disease-specific knowledge. All users recommended Axia to other patients. Conclusion: Axia increases axSpA patients HbE frequency, possibly due to its good intuitiveness and design, leading to reduction in pain and subjective improvement of range of motion. This warrants further investigation in large randomized controlled interventional trials to establish its efficacy conclusively and patients adherence to HbE.

**Supplementary Information:**

The online version contains supplementary material available at 10.1007/s00296-024-05600-w.

## Introduction

Smartphone accessibility has led to the widespread use of mobile exercise apps, with one-third of German adults and half of those under 30 utilizing them according to the W3B-market study by Fittkau and Maaß [1]. In conjunction with this trend, app-based digital therapeutics (DTx) focusing on exercise-related therapy are becoming more integrated into healthcare, particularly for patients needing consistent physiotherapy and exercise routines.

Among rheumatic diseases, axial spondyloarthritis (axSpA), a chronic inflammatory disease of the axial skeleton, has the strongest recommendation for regular disease-specific exercise programs: Exercise therapy is mentioned as one of the two central therapeutic pillars besides pharmacotherapy in national and international guidelines [2, 3]. Besides training in physical therapy facilities and self-help groups, home-based disease-specific exercise programs have shown to improve disease activity (Bath ankylosing spondylitis disease activity index [BASDAI]), disease-specific functionality (Bath ankylosing spondylitis functional index [BASFI]), range of motion (Bath ankylosing spondylitis metrology index [BASMI]) and quality of life (ASQoL [ankylosing spondylitis quality of life], SF-36 [36-item short form survey]) [4–7]. Despite these clear recommendations, a recent survey among 435 axSpA patients of the German patient self-help association “Deutsche Vereinigung Morbus Bechterew” (DVMB) found that nearly 30% of German axSpA-patients exercise too infrequently [8]. This trend has been further exacerbated by the closure of physical therapy facilities, fitness centers, and self-help groups during the SARS-CoV-2 pandemic [8]. In 2019, the European League against rheumatism (EULAR) recommended the use of high-quality and patient-tailored apps for the management of rheumatic diseases [9]. In Germany, a high-quality DTx can be reimbursed by all Statutory Health Insurances and is then called Digital Health application (DHA) (for further details on DHA see our supplementary file) [10]. Although the technical requirements and the wide availability of mobile end devices are given, no German-language DHA providing high-quality and patient-tailored physical therapy specific for axSpA could be established since then.

However, 84% of the participants of the aforementioned survey expressed their wish for such an exercise offering DHA and provided extensive feedback on the essential functionalities an axSpA-focused DHA should include [8]. Beside this, we reviewed different DHA listed in the German DHA directory for other diseases than axSpA revealing that an axSpA-specific DHA might provide further benefits beyond direct therapeutic exercise interventions [10]: We considered additional disease-specific educational information as well as behavioral therapeutic approaches helpful, especially, when addressing important comorbidities of axSpA like insomnia, fatigue, anxiety, or distress [2, 9–14]. Furthermore, features like symptom tracking or medication-reminder functions can improve patients` self-management [9–14].

With these aforementioned facts in mind, we decided to develop a German-language app called “Axia” for people affected by axSpA to close the existing gap in German patient care and to establish the first axSpA-specific DHA [10, 15].

We hypothesize that such a German-language axSpA app can enhance the HbE frequency as well as disease-specific knowledge [4–7, 9, 10]. As part of the development and quality management process, short-term user tests with axSpA patients were mandatory. So, we decided to perform a survey as part of the user tests among these first users of the axSpA app Axia to receive first feedback on functionality, the quality of the exercise programs, and the quality of the information and content before performing larger randomized controlled trials (RCT).

## Methods

### Key functions and development of Axia

**Fig. 1:**
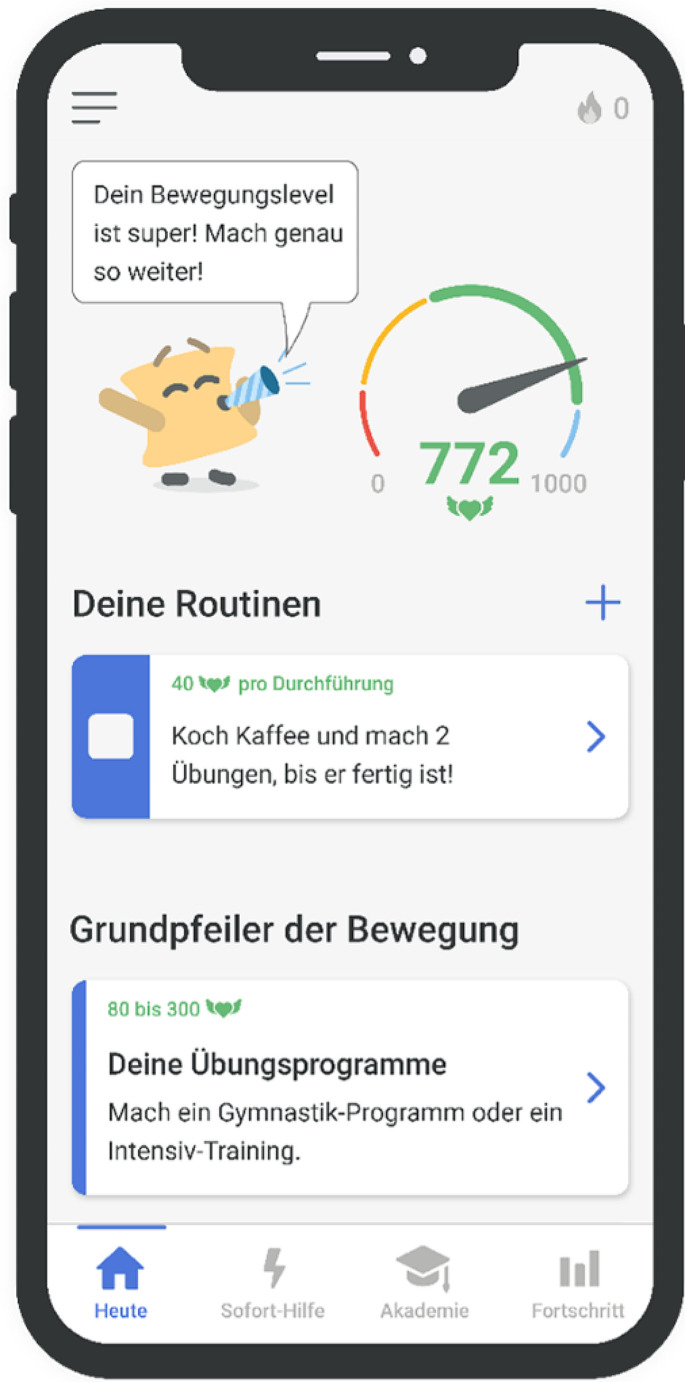
User interface and dashboard of Axia. In the middle of the screen, the main elements of gamification are presented: On the left side, there is the mascot “Bechto” with a green flag expressing positive feedback to the user for high adherence in exercise therapy. On the right side, the “points tank” is shown in the form of a speedometer. Points can be collected by performing disease-specific exercises, sports, or educational lessons. In the middle of the screen, one button for starting an exercise routine and one for starting an exercise program are visible. At the bottom of the screen, you find a menu to navigate between the home screen, acute pain section, educational (Academy), and symptom tracking functions

**Fig. 2:**
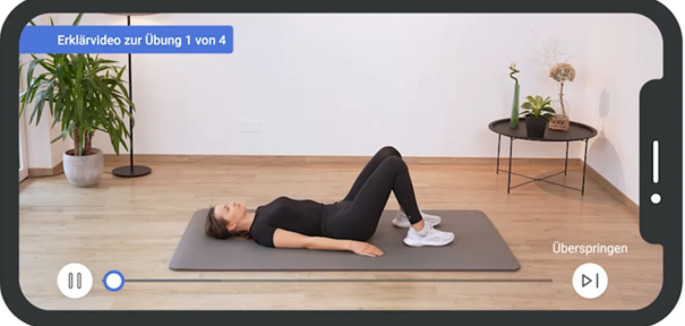
Example of an instructional video. Axia includes over 250 different exercise videos. All of them were professionally filmed with multiple camera angles to present the correct way of exercise execution. Before starting the exercise, an initial instructional video with detailed explanations is presented to the user before starting to perform the exercise itself. A professional narrator guides the patient through the instruction as well as training to ensure correct exercise execution. Experienced users can skip the instructional video and directly perform the exercises

Axia, developed by Applimeda in Aachen, Germany, is a class I medical device, created with inputs from the University Hospital of Würzburg and the DVMB. The app, which is currently available only in German, was informed by market analysis [10, 15], a survey of 435 axSpA patients [8], over 50 axSpA patient interviews, and expert recommendations, ensuring its content and design are closely aligned with user needs and medical guidelines. Figure 1 provides an overview of the user interface. Focused on providing guideline-based exercise therapy, Axia offers personalized exercise programs. The app therefore features over 250 exercise videos (Fig. 2 presents an example of a typical exercise video), a unique learning algorithm for therapy adaptation based on user feedback, and specialized programs for acute pain and intensive training.

The app also serves as an educational resource, offering a knowledge library with 56 interactive articles on axSpA management. Additional features include symptom tracking, a progress dashboard, relaxation exercises, and a medication monitoring function. Our supplementary file provides further information on integrated functions and the development process of Axia.

### Study design

After the above-mentioned development process, the CE marking of Axia as a medical device was achieved in June 2023. Subsequently, user tests with Axia were carried out in the period between 15 July 2023 and 31 October 2023 with the major goals to obtain structured user feedback as well as to collect data on the performance and safety of the app. To use Axia, it was first necessary to download the app from the app store (either Google Play Store or Apple App Store). The testers then created a user account and received an activation code from the manufacturer, which activated the app for a limited period starting from the time when the code was initially entered into the app.

All participants who had access to the app for at least ten days were invited to complete a mixed-method 38-question survey on the app in German language at the end of their usage period. The post-market survey was created *as a web-based survey* using the tool “LamaPoll” (lamapoll.de, Software-As-A-Service) and the data were collected and analysed anonymously [16].

### Patients

Potential participants were informed about the app via DVMB’s own social media channels and the ankylosing spondylitis journal 173 (June 2023) published by the DVMB. Interested persons were invited to contact the manufacturer via email. Inclusion criteria were age over 18 years, diagnosis of axSpA, the possession of a smartphone, sufficient smartphone competence, and the potential to perform at least 30 min gymnastics per day. Exclusion criteria were all conditions not allowing to train daily or perform regular exercise and pregnancy.

Approximately 250 individuals responded. Since the manufacturer planned to include only about 50 axSpA-patients in this quality management process, 54 axSpA-patients were selected chronologically from all enquiries to avoid selection bias and invited to a short video call, during which their app access was explained (activation by entry of an activation code) and any remaining questions were clarified. Participants were each given test access for at least 10 days, with the majority having access to the app for exactly 10 days (n[10d] = 50, n[90d] = 3, n[30d] = 1).

**Fig. 3:**
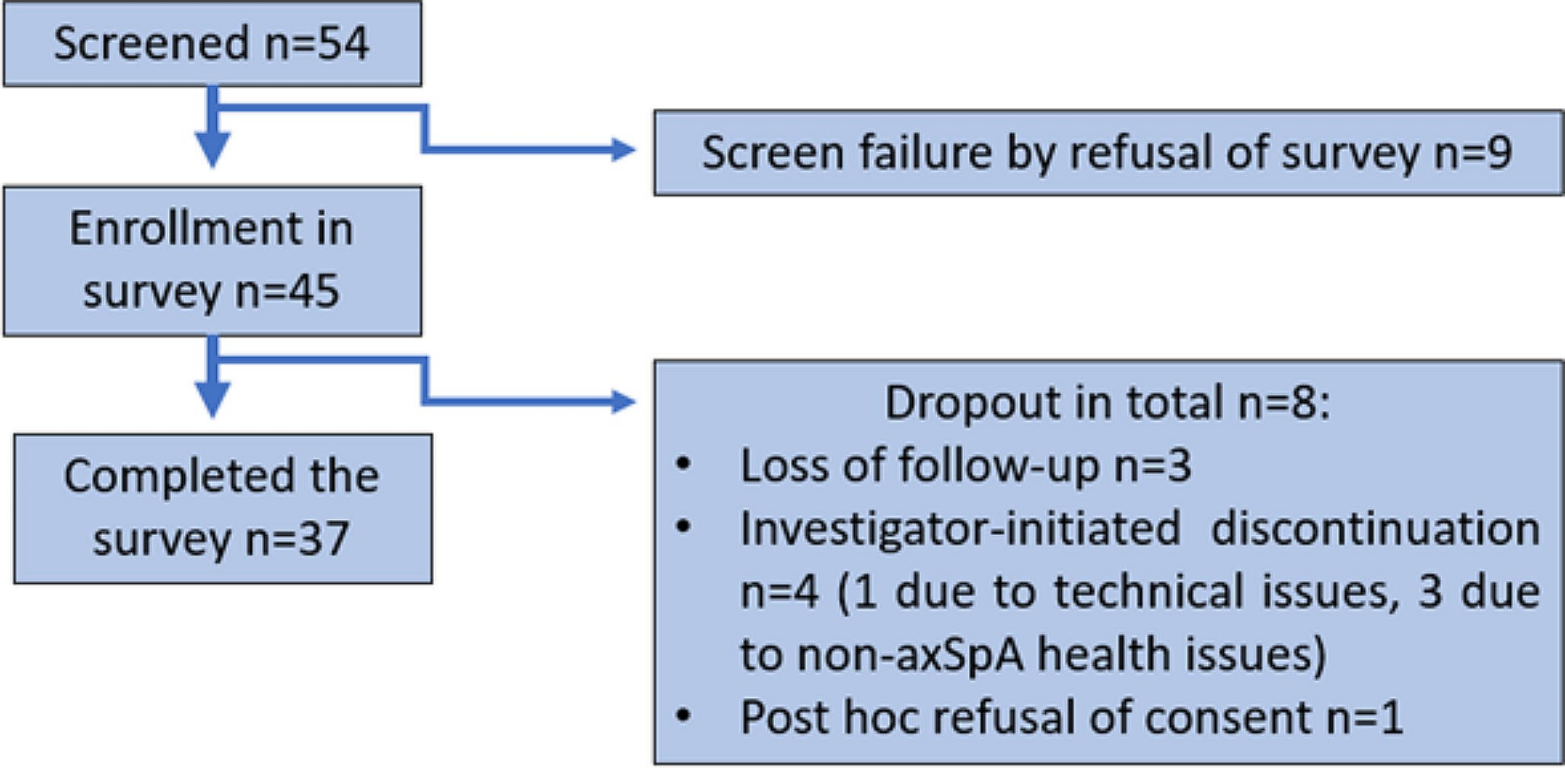
Study flow diagram of the study. All recruited volunteers were able to download Axia from the App Stores and to onboard the App on their mobile end device. Nine participants refused to take part in the survey, from the enrolled subject, four had to be excluded due to non-axSpA-specific medical or technical issues. One participant withdrew his consent to take part in the survey, and three more subjects were lost of follow up in the survey because they did not complete the questionnaire

During the set-up video call, nine of the 54 included people stated that they did not wish to take part in the announced survey. During their usage period, three of the remaining 45 persons reported that they had stopped using the app for other, non-axSpA or app-related medical reasons. They were excluded, just like one other person who stated that he did not wish to provide feedback. Finally, one more user was excluded since he was unable to use the app for technical reasons. Thus, a link to the survey was sent to 40 test users. Out of these, 37 completed the survey and were included in the following analysis (Fig. 3 presents the study flow diagram of the study).

### Questionnaire used in the survey

The survey was planned to ask about various aspects of usage and user experience. Therefore, we designed a questionnaire containing 7 domains (participants characteristics, HbE, design and functionality in general, documentation function, academy function, efficiency, and recommendation to other patients) with 38 self-designed items in total. The translated questionnaire is presented in the supplementary file. The reason for not using the validated standard questionnaires, like BASDAI or user Mobile Application Rating Scale (uMARS), was that we wanted to spare them for the bigger upcoming RCTs [17, 18]. However, the items orientated well on the BASDAI, NET promoter score, uMARS and other validated questionnaires as source [17, 18]. The used items were mainly nominally or ordinally scaled (mainly dichotomous items, numerical rating scales (NRS), or likelihood scales).

### Compliance with ethical standards

This study was performed in line with the principles of the Declaration of Helsinki. All participants provided informed consent to take part in the user tests and the survey. Furthermore, the participants gave informed consent for publication of the data. The study was submitted to the ethics committee of the Medical Faculty of the University of Wuerzburg (DE/EKBY13) (Date December 4th, 2023, No 2023112302). The ethics committee considered the study to be part of the quality management process. Therefore, the study required no further ethical approval according to the national regularities.

### Statistical analysis

LamaPoll provided an Excel file with the anonymous raw data. Then, Excel 2016 was further used for the primary analysis of response frequencies of each item. For correlation studies, SPSS Version 27 was used. Because mainly ordinal-scaled variables were included in the analysis we used the Spearman correlation test. Results were presented as Spearman’s ρ with a two-sided significance test. When *p* < 0.05 results were interpreted as significant, and correlation was interpreted. In accordance with Cohen, ρ > 0.1 was interpreted as low/ weak correlation, ρ > 0.3 as medium, and ρ > 0.5 as large correlation [19]. Continuous values were tested for normal distribution by using the Shapiro-Wilk test via GraphPad Prism, Version 5. Because normal distribution could not be determined, medians with interquartile ranges (IQR) were calculated, and the Mann-Whitney U test was used for analyzing differences. When *p* < 0.05, differences were interpreted as significant.

## Results

### Descriptive information

#### General information

All 37 participants (female = 25, male = 12) who completed the survey had downloaded and activated Axia and set up an individual user account. These people were included in the final analysis (see Fig. 3). They were not given any specifications regarding the frequency of use of the app, nor was their usage behaviour technically checked or tracked after initial activation. There were no reminder emails for users, thus, they were free to use or not to use Axia just as they wanted to.

#### User characteristics

The median age was 50–59 years (IQR 10 years) and the median time since disease onset was 11–20 years (IQR 18–25 years). On an NRS scale (ranging from 0 to 10, while 0 represents no disease activity and 10 the maximum of diseases activity), the median disease activity/ patient global assessment (PtGA) – based on the last four weeks and rated by the participants – was 5 (IQR 3). The median disease-specific pain (NRS pain, ranging from 0 to 10, while 0 represents no pain and 10 the maximum of pain) based on the past four weeks was also 5 (IQR 3). The NRS pain correlated well with the PtGA (ρ = 0.678, *p* < 0.001). Age as well as disease duration did not correlate with PtGA or the NRS pain, but age correlated well with disease duration (ρ = 0.642, *p* < 0.001).

### Evaluation of the user tests

#### Exercise program and HbE frequency with Axia

**Fig. 4:**
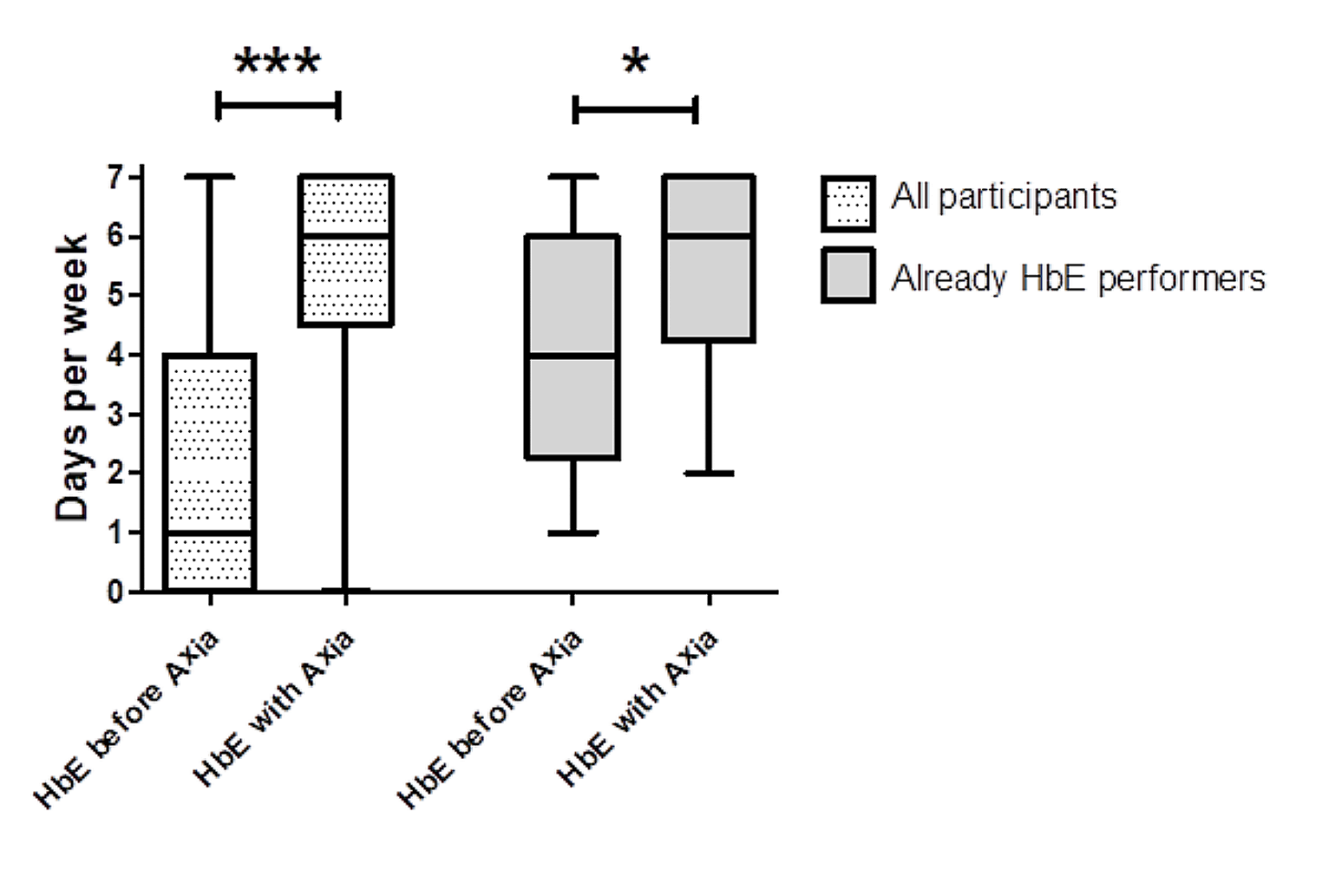
Change of home-based exercise by the use of Axia. The use of Axia significantly improved the frequency of home-based exercised (HbE) compared to the level of the last 4 weeks before start of the study. Only 54.0% (*n* = 20) participants performed regular HbE before the study. Even in this subgroup, the frequency of HbE increased significantly. *: *p* = 0.011; ***: *p* < 0.001

97.3% (*n* = 36) answered that they benefited from the selected exercise program and 91.9% (*n* = 34) agreed that Axia helped them to integrate more exercises into their daily activities. 97.3% (*n* = 36) believed that Axia will keep them training. Before using Axia, 46.0% (*n* = 17) had not performed HbE and with Axia, 97.3% (*n* = 36) performed HbE, thus, Axia increased the total number of new HbE performers by 80% (*n* = 16 subjects started new HbE). However, Axia also increased the quantitative level of HbE: The median of days per week of HbE was 1 (IQR 4) before Axia and increased significantly with the use of Axia to 6 days per week (IQR 2.5, *p* < 0.0001) (Fig. 4). Remarkably, even in the subgroup of participants who had already been performing HbE, the median of days per week performing HbE increased significantly by using Axia (before Axia 4 days per week, IQR 3.75, with Axia 6 days per week, IQR 2.75, *p* = 0.011). In line with this, 56.8% (*n* = 21) answered that they could imagine to train daily with Axia, 37.8% (*n* = 14) would still exercise several times a week, while only 5.4% (*n* = 2) would use Axia only once a week or more seldom in future. 100% (*n* = 37) believe that Axia will help them to achieve or maintain a high level of exercise. A direct correlation was found between the initial NRS pain and the grade of HbE with Axia (ρ = 0.437, *p* = 0.007) as well as the initial PtGA and the grade of HbE with Axia (ρ = 0.409, *p* = 0.012).

#### Design and functionality in general Axia in general

**Fig. 5:**
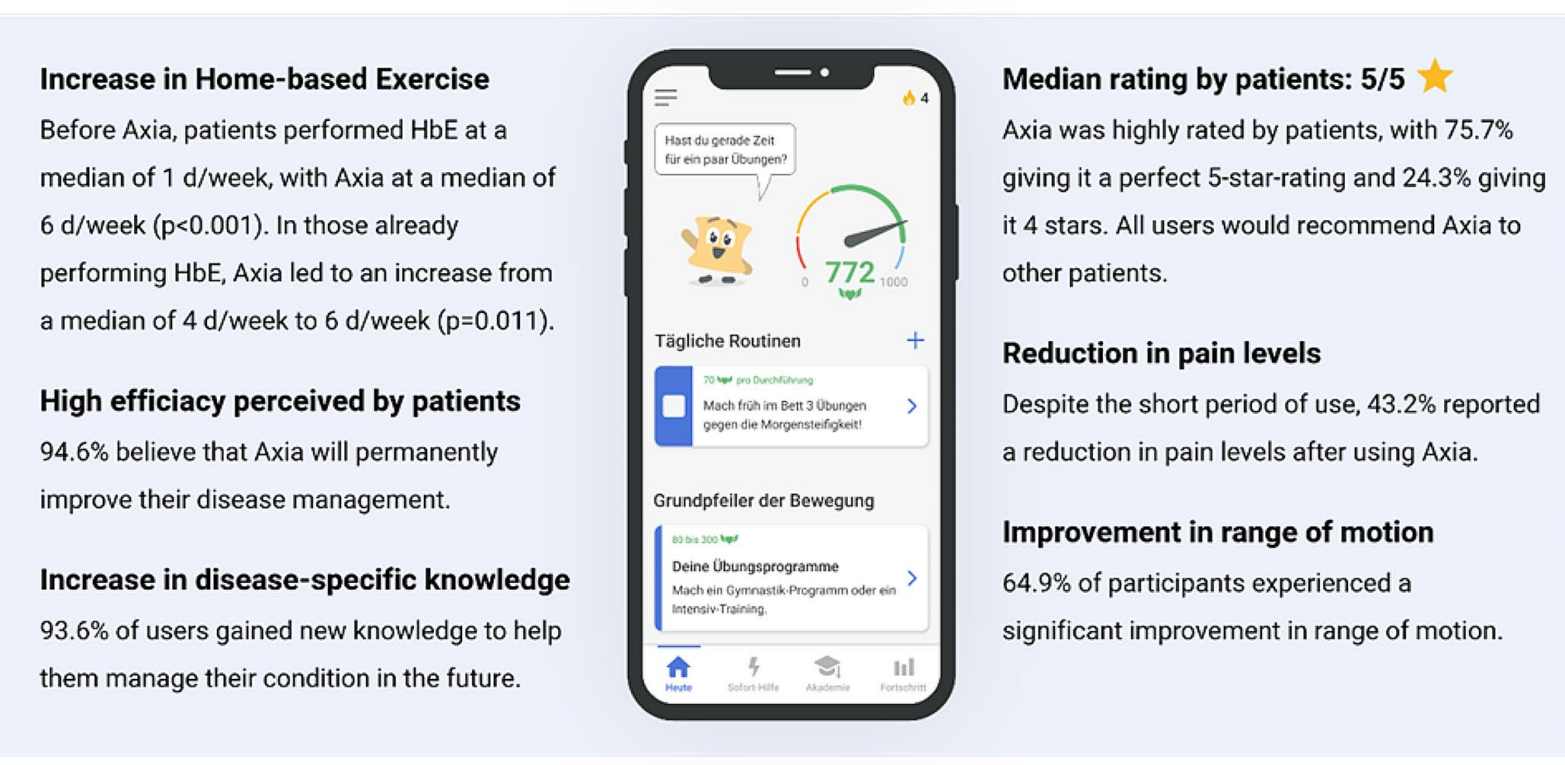
Overview of the user test results of Axia. This figure summarizes the main findings of the user tests and emphasizes that Axia received great ratings by the testers

All users (*n* = 37) could orientate well in Axia and had no issues using all relevant functions. On an NRS (0–10, 0 represents no intuitiveness and 10 the maximum of intuitiveness) to express Axia`s intuitiveness and operability a median NRS of 9 (IQR 2) was achieved. The entertainment factor of Axia was rated on an NRS (0–10, 0 represents no entertainment and 10 the maximum of entertainment) with a median of 8 (IQR 3). Axia`s design and attractiveness were rated with a median of 9 (IQR 2) on an NRS scale (0–10, 0 represents no attractive design and 10 the maximum of attractiveness)). In total, 75.7% (*n* = 28) would rate the app with 5 of 5 stars and 24.3% (*n* = 9) with 4 of 5 stars (median 5 stars, IQR 1). No subject rated Axia worse than 4 stars. 94.6% (*n* = 35) stated that Axia will permanently improve their disease management. 78.4% (*n* = 29) stated that they would be willing to pay for the continued use of Axia. In correlation analysis, the number of awarded stars correlated well with the NRS design (ρ = 0.35, *p* = 0.33) and the satisfaction with Axia in total (ρ = 0.614, *p* = 0.001). Figure 5 summarizes the main results of the user tests.

#### Documentation function

Regarding documentation functions, 100% (*n* = 37) think that Axia is suitable for adequate documentation of sports activities and 86.5% (*n* = 32) stated that Axia is also suitable for documentation of disease-specific symptoms.

#### Academy function

Regarding the patient-educational function, 83.8% (*n* = 31) have used the Academy function at least once, while 16.2%(*n* = 6) have never used it. Of these who used the academy, 93.6% (*n* = 29) are convinced that the academy helped them to improve their disease-specific knowledge, while almost all of them (96.8%, *n* = 30) think that the academy will help them in coping with axSpA. On a scale from 1 to 5 (1 = very bad, 2 = bad, 3 = neutral, 4 = good, 5 = very good), all rated the selection of the covered topics as good (*n* = 14, 45.2%) or even very good (*n* = 17, 54.8%).

#### Subjective disease improvement by using Axia (efficiency)

While 35.1% (*n* = 13) did not notice an improvement in the range of movement, 56.8% (*n* = 21) felt a mild improvement and 8.1% (*n* = 3) even a great improvement, despite the short period of use. Remarkably, no subject stated a worsening of the range of movement. 97.3% (*n* = 36) believe that Axia would improve their flexibility with prolonged use. In correlation analysis, improvement of range of motion correlated well with the grade of HbE with Axia (ρ = 0.432, *p* = 0.008).

Regarding pain, 43.2% (*n* = 16) remarked mild pain relief while 48.7% (*n* = 18) had no improvement of their pain. The residual 8.1% had no pain at all. 89.2% (*n* = 33) think that Axia can help to decrease their level of pain with prolonged use. No significant correlation was found between the grade of pain relief and the grade of HbE with Axia. Interestingly, a direct correlation was found between the initial NRS pain and the subjective pain relief by using Axia (ρ = 0.463, *p* = 0.006).

#### Recommendation to other patients

All of the users would recommend Axia to other patients and 94.6% (*n* = 35) even stated that they were very likely to recommend the app to other patients.

## Discussion

### Principial findings

In this article, the DTx Axia is presented as well as the first data from a patient survey indicating that Axia might be effective for axSpA patients. Besides technical and software issues we also analyzed the results of the survey’s questionnaire: We found that Axia significantly improved the level of HbE independent of the initial level of HbE during the user tests. The fact that DTx can improve patients’ daily activity was also shown before [14, 20, 21]. Thus, Axia clearly fulfilled one of its primary purposes to motivate participants to HbE. Remarkable in this context, we discovered that the NRS pain as well as the PtGA correlated well with the grade of performance of HbE with Axia. This is not surprising, because pain as well as disease activity might be crucial motivating factors for performing disease-specific exercise aiming to improve these impairments in the future. The fact that the majority of the users were willing to exercise with Axia on a daily basis for the most part of their test period might be explained by its appealing design as well as its intuitive use: Axia achieved good user satisfaction, with a median of 5 out of 5 stars awarded to Axia. All users would recommend Axia to others, and almost 78% would even use Axia at their own expense if Axia would not be reimbursed by their health insurance. We believe that we have achieved this high contentment by developing Axia in close cooperation with the axSpA-patients and their patient self-help association DVMB.

### Comparison with other DTx for axSpA

Because establishing of reimbursed DHA with their high-quality standards is a specific German approach, direct comparison of Axia with international DTx is challenging. By reviewing Google Play Store, Apple App Store, and Pubmed, some axSpA-specific apps in Chinese-language could be identified with offering predominantly HbE-motivational interventions in contrast to Axia with HbE as direct intervention [22, 23]. In English language, the Spondylitis Society of America (SSA) offers exercise videos on their homepage without providing an own DTx while the British National Axial Spondyloarthritis Society (NASS) has also published a DTx called “Back to Action” which provides HbE in the form of exercise videos [24, 25].

In contrast, some German-language DTx for axSpA have already been developed : While the majority of already existing rheumatic DTx offers mainly documentary, consulting or patient-educative functions (Pain companion, MyTherapy, AxSpALive, RheumaLive, RheumaBuddy) only a small number of DTx provides direct therapeutic interventions [26]. Here, especially the DTx Rheuma-Auszeit (Rheuma-Liga e.V., Bonn), YogiTherapy (University of Erlangen and University Hospital of Erlangen, Erlangen), Mida Rheuma App (Midaia GmbH, Heidelberg), and reclarit (Gaia, Hamburg and Chugai, Frankfurt/Main) have to be mentioned [14, 27–30]. While reclarit and Mida Rheuma App primarily offer a chatbot-based behavioral therapeutic interventions aiming at improving the quality of life of patients, Rheuma-Auszeit and YogiTherapy also provide exercises for HbE and can therefore be regarded as direct comparison products [14, 27–30]. Rheuma-Auszeit, developed by the German self-help group Rheuma-Liga, is no medical device according to the European MDR and is not specific for axSpA compared to Axia [30]. Nevertheless, Rheuma-Auszeit achieved good results in the uMARS in a validation study with a mixed group of patients suffering from different rheumatic diseases [31]. Furthermore, the license holders of Rheuma-Auszeit (Rheuma-Liga) are not considering to further develop Rheuma-Auszeit into a medical device or even a reimbursed DHA at the moment (according to the information on their homepage [30]). YogiTherapy was developed by the University of Erlangen and offers a Yoga-based training specific for axSpA patients [14, 28]. According to the publication of their user tests, the developers initially planned to develop YogiTherapy as a medical device [14, 28]. So far, YogiTherapy has not yet been certified as a medical device to the best of our knowledge and is now offered as free ware in app stores (as of April 2024). YogiTherapy showed good results in their first user tests regarding attractiveness and stimulation, but these results should be interpreted in the knowledge of the low number of participating patients in their first (*n* = 5) and second survey (*n* = 16) [14, 28]. Furthermore, YogiTherapy mainly offers a Yoga-based exercise form [14, 28]. In conclusion, there is currently no DTx on the German-speaking market similar to Axia which is both a medical device and offers a universal axSpA-specific exercise program.

### How Axia anticipates the challenges for DHA in rheumatic diseases

Only one study has investigated the usage of German-language DHA in rheumatic diseases in the real-world so far and revealed that the main problems of DHAs are on the one hand the onboarding of patients to the DTx and on the other hand to achieve permanent adherence to the DTx [20]. Axia was therefore designed and developed in close collaboration with the affected patients to better address these issues: In our survey, Axia received excellent ratings for its intuitiveness and operability which is crucial for onboarding users. Additionally, all volunteers in our study were able to download and activate Axia successfully. Only one user stopped using Axia later due to technical issues. Thus, we achieved a successful onboarding rate of 100%, which was as double as high as the rate of 46% reported from the study conducted by Labinsky et al. with a similar case number as our survey (*n* = 39) [20]. Our data might of course be confounded by the fact that our participants might have been higher motivated than the subjects in the above-mentioned trial and the onboarding process was further supported by video-calls. Alternatively, Axia might be more attractive for axSpA-patients than the non-rheumatic DHAs which were prescribed by Labinsky and colleagues [20]. Besides the onboarding process, the even more considerable issue of mobile health products is their low adherence rate [20, 32]: Only almost half of the successfully onboarded participants (55%) in the Labinsky trial further used their DHA after the installing process [20]. The phenomenon of fast attrition is also well-known in the whole digital world besides mobile health products [32]. Strikingly, all of our participants in the survey maintained their engagement with Axia and showed an almost daily use of Axia expressed as the median HbE rate of 6 days/week which overwhelms the adherence rates reported by Labinsky et al. [20]. We believe that this might at least partly be caused by the gamification factor and feedback mechanisms of Axia leading to its good ratings for design as well as for entertainment. Of course, our observational period of almost two weeks was very short compared to the three months in the Labinsky trial [20]. Nevertheless, a considerable proportion of users in studies stop using their apps and mobile health products shortly after beginning to use them, which is comparable to our observational period [20, 32]. We were unable to observe this high attrition rate, as only four users in our study stopped using Axia. In line with this, 97% of the participants of the survey stated that they would use Axia permanently in future. Whether Axia permanently leads to such high adherence rates in the long term must be examined in further studies with longer observation periods.

### Strength and limitations

The findings of our study must be interpreted with caution due to its several limitations:

i. Small Sample Size: The study had a limited sample size of 37 participants, and all data was self-reported by patients.
ii. Questionnaire Selection: We avoided using extensive but validated questionnaires like uMARS or BASDAI to reduce the burden on participants. These will be employed in future randomized controlled trials.
iii. Volunteer Participants: All participants were volunteers from the German self-help association DVMB, potentially representing a highly motivated subgroup more likely to adopt new therapies than the general axSpA patient population in Germany. To avoid a selection bias, we did not pick the most suitable or motivated participants but recruited chronologically by the time of the email receiving.
iv. Unverified User Statements: We did not verify the accuracy of users’ statements about their frequency of app usage.
v. High Screen Failure and Dropout Rates: It remains unclear why a high proportion (nine of 54/ 17%) of the screened subjects did not want to take part in the survey. Eight of 45 enrolled subjects (18%) dropped out due to several reasons. Due to the design, the analysis could only be carried out with the per protocol approach. It is possible that only the most motivated and fittest subjects completed the study, with possible distortion of the results.
vi. Preliminary Data: Despite these limitations, this study provides initial insights into the use, functionality, and user satisfaction of a new digital medical device. It highlights a novel form of digital exercise intervention highly sought after by German axSpA patients [8].

These factors underline the need for further research with a broader and more diverse participant base and longer observation periods to validate these initial findings.

### Conclusions and future steps

In conclusion, Axia is a new CE-marked medical device offering a digital form of patient-tailored exercise intervention for patients with axSpA. Axia helped users to increase their HbE frequency, regardless of prior engagement in HbE activities, possibly due to its high user satisfaction and recommendation rates in user testing. The next phase involves conducting two large RCTs with over 200 axSpA patients to evaluate Axia’s efficacy and adherence rates over time. These studies started in March 2024 (DRKS-ID 00033783), aiming to establish the first DHA for axSpA in the German healthcare system and to provide a new, validated method of exercise intervention.

### Electronic supplementary material

Below is the link to the electronic supplementary material.

Supplementary Material 1

## Data Availability

The source data that support the findings of this study are not openly available due to reasons of sensitivity. Upon reasonable request, these date are available from the corresponding author. Data are located in controlled access data storage at University Hospital of Wuerzburg.

## Abbreviations

App: application
axSpA: axial Spondyloarthritis
ASQoL: Ankylosing spondylitis quality of life
BASDAI: Bath ankylosing spondylitis disease activity index
BASFI: Bath ankylosing spondylitis functional index
BASMI: Bath ankylosing spondylitis metrology index
CE: Conformité Européenne
DHA: digital health application (for further information regarding the difference between DTx and DHA see our supplementary file)
DTx: digital therapeutics
DVMB: Deutsche Vereinigung Morbus Bechterew (German self-help association for axSpA)
HbE: home-based exercise
MDR: medical device regulation
SF-36: 36-item short form survey
