# Supplementary material for: The exercise-app Axia for axial spondyloarthritis enhances the home-based exercise frequency in axial spondyloarthritis patients – A cross-sectional survey
Source: Rheumatol Int. 2024 Apr 29;44(6):1143–54. doi: 10.1007/s00296-024-05600-w (PMC11108939; doi:10.1007/s00296-024-05600-w)
Supplement: Supplementary file 1 — Supplementary Material 1 [file 296_2024_5600_MOESM1_ESM.docx]

**Supplementary file to “The Exercise-App Axia for axial Spondyloarthritis enhances the Home-based exercise frequency in axial Spondyloarthritis patients – A cross-sectional survey.”**

**Authors**

Patrick-Pascal Strunz* ^1^, ORCID: 0000-0002-3168-7074,

Maxime Le Maire* ^2^, ORCID: 0009-0000-1202-8447,

Tobias Heusinger ^2^, ORCID: 0009-0002-8883-3404,

Juliana Klein ^2^, ORCID: 0009-0009-4941-2683,

Hannah Labinsky ^1^, ORCID: 0000-0001-5762-9182,

Anna Fleischer ^3^, ORCID: 0009-0009-8760-3931,

Karsten Sebastian Luetkens ^4^, ORCID: 0000-0001-7536-9409,

Patricia Possler ^2^, ORCID: 0009-0007-1773-4763,

Michael Gernert ^1^, ORCID: 0000-0003-0074-4459,

Robert Leppich ^5^, ORCID: 0000-0003-4711-7743,

Astrid Schmieder ^6^, ORCID: 0000-0002-6421-9699,

Ludwig Hammel ^7^, ORCID: 0009-0003-8403-0652,

Evelin Schulz ^7^, ORCID: 0009-0002-8116-4308,

Billy Sperlich ^8^, ORCID: 0000-0003-4686-8561,

Matthias Froehlich ^1^, ORCID: 0000-0001-7745-3903,

Marc Schmalzing^1^, ORCID: 0000-0002-3289-2299.

**Affiliations**

^1^ University Hospital Würzburg, Department of Internal Medicine 2, Rheumatology/Clinical Immunology, Oberdürrbacher Straße 6, 97080 Würzburg, Germany

^2^ University of Würzburg, Medical Faculty, Josef-Schneider-Straße 2, 97080 Würzburg, Germany

^3^ University Hospital Würzburg, Department of Internal Medicine 2, Psychosomatic Medicine, Oberdürrbacher Straße 6, 97080 Würzburg, Germany

^4^ University Hospital Würzburg, Department of Diagnostic and Interventional Radiology, Oberdürrbacher Straße 6, 97080 Würzburg, Germany

^5^ Chair of Software Engineering (Informatik II), Department of Computer Science, University of Würzburg, Am Hubland, 97074 Würzburg, Germany

^6^ University Hospital Würzburg, Department of Dermatology, Venereology, and Allergology, Josef-Schneider-Straße 2, 97080 Würzburg, Germany

^7^ Deutsche Vereinigung Morbus Bechterew e. V., Metzgergasse 16, 97421 Schweinfurt, Germany.

^8^ Integrative and Experimental Exercise Science and Training, Institute for Sports Science, University of Wuerzburg. Judenbühlweg 11, 97082 Würzburg

* Contributed equally

**Corresponding author**

Dr. med. Patrick-Pascal Strunz,

University Hospital Würzburg,

Department of Internal Medicine 2, Rheumatology/Clinical Immunology,

Oberdürrbacher Straße 6,

97080 Würzburg, Germany

Email: [Strunz_P@ukw.de](mailto:Strunz_P@ukw.de)

**1. Questionnaire**

| **Section I: Participants Characteristics** | | | | | | | | | | |
| --- | --- | --- | --- | --- | --- | --- | --- | --- | --- | --- |
| 1. How old are you? | | | | | | | | | | |
| 18-29  30-39  40-49  50-59  60-69  70 or older | | | | | | | | | | |
| 1. How long have you been diagnosed with axSpA or ankylosing spondylitis? | | | | | | | | | | |
| under 1 year  1 - 2 years  3 - 5 years  6 - 10 years  11 - 20 years  21 - 30 years  31 years or longer | | | | | | | | | | |
| 1. On a scale of 0-10 (0 no pain, 10 maximum imaginable pain), how would you rate the severity of your axSpA or ankylosing spondylitis-related pain in the last 4 weeks before using the app? (NRS pain) | | | | | | | | | | |
| 0 | 1 | 2 | 3 | 4 | 5 | 6 | 7 | 8 | 9 | 10 |
| 1. On a scale of 0-10 (0 no activity, 10 maximum imaginable activity), how would you rate the disease activity of your ankylosing spondylitis or axSpA within the last 4 weeks before using the app? (Patient Global activity) | | | | | | | | | | |
| 0 | 1 | 2 | 3 | 4 | 5 | 6 | 7 | 8 | 9 | 10 |
| **Section II: Home-based Exercise** | | | | | | | | | | |
| 1. The Axia app offers you customized exercise programs with video instructions. Can you imagine exercising regularly in the future under the guidance of the app? | | | | | | | | | | |
| Yes, daily  Yes, several times a week  Yes, once or less per week  No | | | | | | | | | | |
| 1. Do you think Axia would help you build or maintain a high level of physical activity in the future? | | | | | | | | | | |
| Yes  No | | | | | | | | | | |
| 1. Do you feel that the exercises in Axia have helped you? | | | | | | | | | | |
| Yes  No | | | | | | | | | | |
| 1. Has Axia helped you to integrate more exercises into your everyday life? | | | | | | | | | | |
| Yes  No | | | | | | | | | | |
| 1. Do you think Axia could help you to perform your exercises more consistently in the long term? | | | | | | | | | | |
| Yes  No | | | | | | | | | | |
| 1. Did you perform specific exercises for your axial spondyloarthritis independently at home in the last 4 weeks before using the app? | | | | | | | | | | |
| Yes  No | | | | | | | | | | |
| 1. If yes, on how many days a week on average? | | | | | | | | | | |
| Free text | | | | | | | | | | |
| 1. If yes, how many minutes in total per week (on average)? | | | | | | | | | | |
| Free text | | | | | | | | | | |
| 1. In the last 10 days while using the app, have you independently performed specific exercises for your axial spondyloarthritis at home? | | | | | | | | | | |
| Yes  No | | | | | | | | | | |
| 1. If yes, on how many days a week on average? | | | | | | | | | | |
| Free text | | | | | | | | | | |
| 1. If yes, how many minutes in total per week (on average)? | | | | | | | | | | |
| Free text | | | | | | | | | | |
| **Section III: Design and Functionality in General** | | | | | | | | | | |
| 1. Have you found your way around the app? | | | | | | | | | | |
| Yes  No | | | | | | | | | | |
| 1. On a scale of 0-10 (0 no intuitive, 10 maximum imaginable intuitiveness), how easy is it to learn how to use the app? How clear are the names of the menus, icons and instructions? | | | | | | | | | | |
| 0 | 1 | 2 | 3 | 4 | 5 | 6 | 7 | 8 | 9 | 10 |
| 1. On a scale of 0-10 (0 no entertainment, 10 maximum imaginable entertainment), how entertaining do you think Axia is to use? | | | | | | | | | | |
| 0 | 1 | 2 | 3 | 4 | 5 | 6 | 7 | 8 | 9 | 10 |
| 1. On a scale of 0-10 (0 not attractive, 10 maximum imaginable attractiveness), how do you like the design of the app? | | | | | | | | | | |
| 0 | 1 | 2 | 3 | 4 | 5 | 6 | 7 | 8 | 9 | 10 |
| 1. How many times would you use the app in the next 12 months if you had unlimited access to it? | | | | | | | | | | |
| Never  1-2  3-10  10-50  More than 50 times | | | | | | | | | | |
| 1. How many stars would you give the app overall? | | | | | | | | | | |
| *  **  ***  ****  ***** | | | | | | | | | | |
| 1. How would you rate your overall Axia experience? | | | | | | | | | | |
| Very happy  Happy  Neutral  Unhappy  Very unhappy | | | | | | | | | | |
| 1. How likely is it that you will ask for access to Axia again once Axia is available free of charge to all patients as an "app on prescription"? | | | | | | | | | | |
| Very likely  Likely  Neutral  Unlikely  Very unlikely | | | | | | | | | | |
| 1. Would you use Axia even if you had to pay for it? | | | | | | | | | | |
| Yes  No | | | | | | | | | | |
| 1. On a scale of 1-5 (1= very important, 2= important, 3= neutral, 4= unimportant, 5= very unimportant), how important are the following contents of Axia to you? | | | | | | | | | | |
| Academy  Daily routines/ habits  Drug monitoring  Relaxing exercises  Daily Exercises  Intensive training function  Motivating functions  Symptom tracking  Acute support for pain  Acute support for morning stiffness  Pedometer  Documentation of progress | | | | | | | | | | |
| **Section IV: Documentation Function** | | | | | | | | | | |
| 1. In your opinion, is Axia suitable for documenting sports or activities? | | | | | | | | | | |
| Yes  No | | | | | | | | | | |
| 1. In your opinion, is Axia suitable for recording and documenting symptoms? | | | | | | | | | | |
| Yes  No | | | | | | | | | | |
| **Section V: Academy Function** | | | | | | | | | | |
| 1. Have you used the academy function in Axia? | | | | | | | | | | |
| Yes  No | | | | | | | | | | |
| 1. Have you been able to acquire knowledge by using the academy in Axia that has helped you or is likely to help you in the future? | | | | | | | | | | |
| Yes  No | | | | | | | | | | |
| 1. Do you think that the learning units offered at the academy can help you to cope better with the disease? | | | | | | | | | | |
| Yes  No | | | | | | | | | | |
| 1. What did you think of the selection of topics in the Academy of the App? | | | | | | | | | | |
| Very good  Good  Neutral  Bad  Very bad | | | | | | | | | | |
| 1. How helpful did you find the learning units offered in the app's academy? | | | | | | | | | | |
| Very helpful  Helpful  Less helpful  Not helpful | | | | | | | | | | |
| **Section VI: Efficiency** | | | | | | | | | | |
| 1. Do you think Axia can help you to cope better with the disease? | | | | | | | | | | |
| Yes  No | | | | | | | | | | |
| 1. Have you already noticed an improvement in your range of movement by using the app? | | | | | | | | | | |
| Yes significant improvement  Yes mild improvement  No improvement  Rather deterioration | | | | | | | | | | |
| 1. Have you already noticed a reduction in your pain through the app? | | | | | | | | | | |
| Yes significant improvement  Yes mild improvement  No improvement  Rather deterioration  I have no pain at all | | | | | | | | | | |
| 1. Do you think that you could reduce your pain in the long term by using Axia? | | | | | | | | | | |
| Yes  No  I have no pain at all | | | | | | | | | | |
| 1. Do you think that you could improve or better maintain your range of motion in the long term by using Axia? | | | | | | | | | | |
| Yes  No | | | | | | | | | | |
| **Section VII: Recommendation to other Patients** | | | | | | | | | | |
| 1. How likely is it that you would recommend Axia to someone else? | | | | | | | | | | |
| Very likely  Likely  Neutral  Unlikely  Very unlikely | | | | | | | | | | |

**2. The difference between a DTx and a DHA**

Three years ago, a novel approach was introduced in Germany, which has since received increasing international attention: In the German healthcare system, innovative digital therapeutics (DTx) that demonstrate a clinical benefit in patient care, are labeled as medical device according to the European Medical Device Regulation (MDR), and pass a central approval process can be prescribed by physicians, similar to medication, and are eligible for reimbursement by all Statutory Health Insurances. DTx successfully passing this approval process are known as "Digital Health Applications" (DHA, or "Digitale Gesundheitsanwendungen" [DIGA] in German) and are included in an exclusive directory, the "German DIGA Verzeichnis," managed by the German authorities (DHA directory) [1]. Being listed in this directory is a mandatory requirement for a DTx to be reimbursed by all health insurance companies.

**3. Axia Design, Content and Development**

**3.1. Development process and Design of Axia**

Axia is a class I medical device that was developed by Applimeda (Aachen, Germany), a startup founded by medical students, in close cooperation with the University Hospital of Würzburg and the DVMB.

The app’s design and content are based on a market analysis of other approved DHAs [1], the results of a survey among 435 axSpA patients [2], individual interviews with more than 50 patients, and recommendations from rheumatologists at the University Hospital of Würzburg. In addition, several physical therapists specialized in axSpA were closely involved in the development of all exercise programs. To make Axia available for both iOS and Android devices, the cross-platform framework Flutter was used. So far, Axia is only available in German language. Figure 1 gives an overview of the user interface.

**3.2. Providing guideline-based exercise therapy**

Axia does not only offer individually tailored exercise programs for axSpA-patients, but also encourages them to adopt short daily exercise habits, engage in sports, and achieve a daily step goal. This promotes a holistic approach to exercise therapy and an active and sporty lifestyle in line with the guidelines [3, 4].

The core of the Axia app features a variety of exercise programs offering exercise suggestions tailored to the patient's flexibility, pain regions, age, and already existing movement restrictions. Axia includes a unique learning algorithm that continuously adapts the therapy to patient feedback by individually selecting optimal exercises from a comprehensive training catalog. For this purpose and under the guidance of specialized physical therapists, more than 250 exercise videos were professionally produced using multiple camera angles to present the correct way of exercise performance. In an initial instructional video, the patient is first given an explanation of each exercise. Afterward, the narrator guides the patient through the exercise while providing additional coaching to ensure correct execution. Figure 2 presents an example of a typical exercise video.

Besides individualized exercise programs designed to maintain flexibility, upright posture, and pain relief over the long term, Axia also offers tailored exercises for acute pain and morning stiffness based on the patient's current pain location and aggregated data, with the goal of providing quick relief to the patient in painful situations. In additional “intensive training” sessions, the user can further choose exercise programs with higher intensity for also addressing cardio-fitness.

To further promote physical activity in everyday life, Axia encourages patients to incorporate small exercise habits into their daily routine. Using a pedometer integrated into Axia, the patients can track a self-imposed step target using their smartphones or a wearable as a sensor. Finally, Axia offers a documentation function for sports activities and physiotherapy sessions.

**3.3. Motivation and Gamification to achieve long-term adherence**

Maintaining a high level of patient adherence is one of the most important and challenging factors for the success of DTx [5]. For this reason, Axia not only focuses on proven methods of motivational psychology, such as the acquisition of long-lasting habits, but also on a gamification concept for the app with a rewarding and individualized points system for exercise therapy activities. In addition, an animated mascot named “Bechto” motivates the patients with humorous animations and encouraging messages. Patients are also motivated to achieve milestones and streaks, completing the gamification concept of the app. A notification system reminds patients of their exercise, especially when they are at risk of missing their exercise goals (The mascot as well as the “points tank” are presented in Figure 1).

**3.4. Information on the disease and axSpA management**

In a knowledge library with 56 interactive learning articles, the patients receive information on important disease-related topics and how to optimize daily life with axSpA. The library is divided into courses, each containing between two and nine interactive learning units. Courses cover a broad range of topics, such as explanations of the clinical presentations and therapies, the importance of exercise for axSpA patients, improvement of sleep quality, management of disease flares, information on medication and nutrition, and other related topics. There is also a dedicated course that provides the most essential information for newly diagnosed patients.

**3.5. Symptom tracking, progress dashboard and other features**

Axia allows patients to make regular symptom queries including pain levels, patient global assessment (PGA), and stress levels. On a progress tracker dashboard, patients can see the development of their symptoms as well as their activity level in Axia for different time frames (last 7/30 days and last 3/6/12 months). Patients can also export a progress report as a PDF file. To assist patients in managing difficult situations and learning relaxation techniques, a variety of relaxation exercises are available, such as progressive muscle relaxation, autogenic training, and brief relaxation exercises. Patients can also monitor their medication using the medication function.

**4. References**

1. Bundesinstitut für Arzneimittel und Medizinprodukte (BfArM) (2023) DIGA Verzeichnis [DHA directory]. Bundesinstitut für Arzneimittel und Medizinprodukte (BfArM). https://diga.bfarm.de/de/verzeichnis. Accessed 12 November 2023

2. Strunz PP, Maire ML, Heusinger T et al (2021) Apps in rheumatology: Is there a need for an app in therapy for axial spondyloarthritis? Z Rheumatol 82(3):256-261. German. doi: 10.1007/s00393-021-01104-1. Epub 2021 Oct 7. Erratum in: Z Rheumatol. 2021 Nov 8;: PMID: 34618212; PMCID: PMC10076400

3. Kiltz U, Braun J, DGRh et al (2019) Langfassung zur S3-Leitlinie Axiale Spondyloarthritis inklusive Morbus Bechterew und Frühformen, Update 2019 : Evidenzbasierte Leitlinie der Deutschen Gesellschaft für Rheumatologie (DGRh) und der beteiligten medizinisch-wissenschaftlichen Fachgesellschaften und weiterer Organisationen [Long version on the S3 guidelines for axial spondyloarthritis including Bechterew's disease and early forms, Update 2019 : Evidence-based guidelines of the German Society for Rheumatology (DGRh) and participating medical scientific specialist societies and other organizations]. Z Rheumatol 78(Suppl 1):3-64. German. doi: 10.1007/s00393-019-0670-3. PMID: 31784900

4. Ramiro S, Nikiphorou E, Sepriano A et al (2023) ASAS-EULAR recommendations for the management of axial spondyloarthritis: 2022 update. Ann Rheum Dis 82(1):19-34. doi: 10.1136/ard-2022-223296. Epub 2022 Oct 21. PMID: 36270658

5. Druce KL, Dixon WG, McBeth J (2019) Maximizing Engagement in Mobile Health Studies: Lessons Learned and Future Directions. *Rheum Dis Clin North Am 45(2):159-172. doi: 10.1016/j.rdc.2019.01.004. Epub 2019 Mar 8. PMID: 30952390; PMCID: PMC6483978.*
